# *catena*-Poly[[tris­(pyridine-κ*N*)copper(II)]-μ-tetra­fluoro­terephthalato-κ^2^ *O* ^1^:*O* ^4^]

**DOI:** 10.1107/S1600536808018977

**Published:** 2008-06-28

**Authors:** Chang-Ge Zheng, Jie Zhang, Jian-Quan Hong, Song Li

**Affiliations:** a School of Chemical and Materials Engineering, Jiangnan University, 1800 Lihu Road, Wuxi, Jiangsu 214122, People’s Republic of China

## Abstract

In the title compound, [Cu(C_8_F_4_O_4_)(C_5_H_5_N)_3_]_*n*_, the Cu^II^ atom, lying on a twofold rotation axis, is five-coordinated by two O atoms from two tetra­fluoro­terephthalate ligands and three N atoms from three pyridine ligands in a distorted trigonal-bipyramidal geometry. Adjacent Cu^II^ atoms are connected *via* the bridging tetra­fluoro­terephthalate ligands into a one-dimensional chain running along the [101] direction.

## Related literature

For related literature, see: Baeg & Lee (2003 ▶); Baruah *et al.* (2007 ▶); Bastin *et al.* (2008 ▶); Cheng *et al.* (2007 ▶); Eddaoudi *et al.* (2000 ▶); Gould *et al.* (2008 ▶); Reineke *et al.* (1999 ▶); Stephenson & Hardie (2006 ▶); Yuan *et al.* (2004 ▶); Zhang *et al.* (2007 ▶); Zheng *et al.* (2008 ▶).
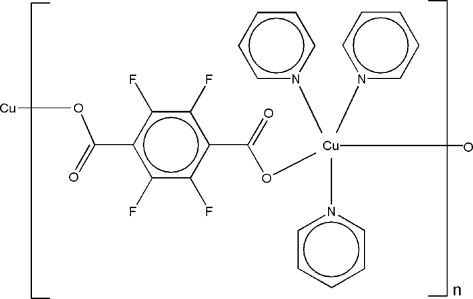


## Experimental

### 

#### Crystal data

[Cu(C_8_F_4_O_4_)(C_5_H_5_N)_3_]
*M* *_r_* = 536.92
Monoclinic, 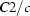

*a* = 15.3579 (8) Å
*b* = 8.7652 (5) Å
*c* = 16.6050 (9) Å
β = 100.241 (3)°
*V* = 2199.7 (2) Å^3^
*Z* = 4
Mo *K*α radiation
μ = 1.06 mm^−1^
*T* = 273 (2) K
0.15 × 0.10 × 0.06 mm

#### Data collection

Bruker SMART APEXII CCD area-detector diffractometer
Absorption correction: multi-scan (*SADABS*; Sheldrick, 1996 ▶) *T*_min_ = 0.857, *T*_max_ = 0.939
10049 measured reflections
1950 independent reflections
1857 reflections with *I* > 2σ(*I*)
*R*_int_ = 0.022

#### Refinement

*R*[*F* ^2^ > 2σ(*F* ^2^)] = 0.025
*wR*(*F* ^2^) = 0.071
*S* = 1.09
1950 reflections
160 parameters
H-atom parameters constrained
Δρ_max_ = 0.29 e Å^−3^
Δρ_min_ = −0.32 e Å^−3^

### 

Data collection: *APEX2* (Bruker, 2007 ▶); cell refinement: *SAINT* (Bruker, 2007 ▶); data reduction: *SAINT*; program(s) used to solve structure: *SIR97* (Altomare *et al.*, 1999 ▶); program(s) used to refine structure: *SHELXL97* (Sheldrick, 2008 ▶); molecular graphics: *PLATON* (Spek, 2003 ▶); software used to prepare material for publication: *SHELXL97*.

## Supplementary Material

Crystal structure: contains datablocks global, I. DOI: 10.1107/S1600536808018977/hy2139sup1.cif

Structure factors: contains datablocks I. DOI: 10.1107/S1600536808018977/hy2139Isup2.hkl

Additional supplementary materials:  crystallographic information; 3D view; checkCIF report

## Figures and Tables

**Table:** 

Cu1—N1	2.0236 (16)
Cu1—O1	2.0609 (12)
Cu1—N2	2.073 (2)

**Table:** 

N1—Cu1—N1^i^	174.71 (9)
N1—Cu1—O1	91.84 (6)
N1—Cu1—O1^i^	85.17 (6)
O1—Cu1—O1^i^	111.11 (8)
N1—Cu1—N2	92.64 (4)
O1—Cu1—N2	124.45 (4)

Symmetry code: (i) 
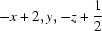
.

**Fig. 1.**
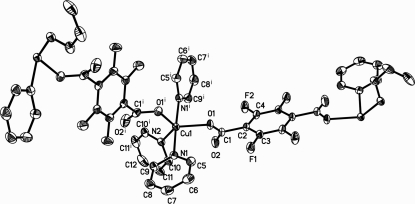
A portion of the chain structure of the title compound. Displacement ellipsoids are drawn at the 30% probability level. H atoms have been omitted for clarity. [Symmetry code: (i) 2-x, y, 1/2-z.)

**Fig. 2.**
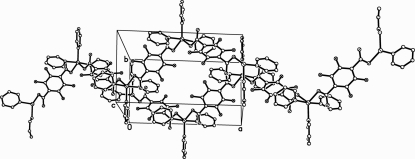
View of the unit cell of the title compound.

## supplementary crystallographic information

### Comment

Recently, organically directed metal–terephthalates have attracted much attention due to their novel structures and desirable physical properties, and a lot of research work has been done on this type of complexes (Bastin *et al.*,  2008; Eddaoudi *et al.*,  2000; Gould *et al.*,  2008). However, there are rare reports about halogen substituted terephthalate metal complexes till now. Some research work in computational study suggests that adsorption property in gas storage can be improved with electronegative atoms (*e.g.* halogen atoms) in the organic linkers or frameworks (Zhang *et al.*,  2007). New topologies with favorable properties will be achieved by introducing some strong electronegative atoms to the phenyl ring.

The title compound consists of one-dimensional neutral zig-zag chains (Fig. 1 and Fig. 2). The tetrafluoroterephthalate ligand is coordinated to Cu^II^ ion in a bridging bis-monodentate fashion. In the trigonal bipyramidal coordination unit, two O atoms from two tetrafluoroterephthalate ligands and one N atom from a pyridine molecule form the equatorial plane. The axial positions are occupied by N atoms from two pyridine molecules with an N—Cu—N angle of 174.71 (9)° (Table 1). The Cu—N bond lengths lie in the range of 2.0236 (16) to 2.073 (2) Å and agree well with the reported values (Baruah *et al.*,  2007; Cheng *et al.*,  2007). The Cu—O bond lengths are 2.0609 (12) Å, which are comparable with the reported values in the similar complexes (Baeg & Lee, 2003; Stephenson & Hardie, 2006; Yuan *et al.*,  2004). In the aromatic ring systems, the values of bond lengths and angles coincide with those previously reported (Zheng *et al.*,  2008).

### Experimental

All the reagents and solvents employed were commercially available. Tetrafluoroterephthalic acid was purified by recrystallization. According to the literature procedure (Reineke *et al.*,  1999), the title compound was synthesized by slow vapor diffusion at room temperature of pyridine (3 ml) into an *N*,*N*-dimethylformamide solution (2 ml) containing a mixture of tetrafluoroterephthalic acid (0.071 g, 0.30 mmol) and Cu(NO_3_)_2_.3H_2_O (0.036 g, 0.15 mmol) diluted with CH_3_OH (6 ml). After two weeks, blue block-shaped crystals were obtained (yield 55% based on Cu). Analysis, calculated for C_23_H_15_CuF_4_N_3_O_4_: C 51.45, H 2.82, N 7.82%; found: C 51.50, H 2.86, N 7.76%.

### Refinement

H atoms were positioned geometrically and refined as riding atoms, with C—H = 0.93 Å and *U*_iso_(H) = 1.2*U*_eq_(C).

### Crystal data

**Table:**

[Cu(C_8_F_4_O_4_)(C_5_H_5_N)_3_]	*F*_000_ = 1084
*M**_r_* = 536.92	*D*_x_ = 1.621 Mg m^−^^3^
Monoclinic, *C*2/*c*	Mo *K*α radiation λ = 0.71073 Å
Hall symbol: -C 2yc	Cell parameters from 7406 reflections
*a* = 15.3579 (8) Å	θ = 2.7–28.3º
*b* = 8.7652 (5) Å	µ = 1.06 mm^−^^1^
*c* = 16.6050 (9) Å	*T* = 273 (2) K
β = 100.241 (3)º	Block, blue
*V* = 2199.7 (2) Å^3^	0.15 × 0.10 × 0.06 mm
*Z* = 4	

### Data collection

**Table:**

Bruker SMART APEXII CCD area-detector diffractometer	1950 independent reflections
Radiation source: fine-focus sealed tube	1857 reflections with *I* > 2σ(*I*)
Monochromator: graphite	*R*_int_ = 0.022
*T* = 273(2) K	θ_max_ = 25.1º
φ and ω scans	θ_min_ = 2.5º
Absorption correction: multi-scan(SADABS; Sheldrick, 1996)	*h* = −18→18
*T*_min_ = 0.857, *T*_max_ = 0.939	*k* = −10→10
10049 measured reflections	*l* = −19→19

### Refinement

**Table:**

Refinement on *F*^2^	H-atom parameters constrained
Least-squares matrix: full	*w* = 1/[σ^2^(*F*_o_^2^) + (0.076*P*)^2^ + 0.2195*P*], *P* = (*F*_o_^2^ + 2*F*_c_^2^)/3
*R*[*F*^2^ > 2σ(*F*^2^)] = 0.025	(Δ/σ)_max_ < 0.001
*wR*(*F*^2^) = 0.071	Δρ_max_ = 0.29 e Å^−^^3^
*S* = 1.09	Δρ_min_ = −0.32 e Å^−^^3^
1950 reflections	Extinction correction: none
160 parameters	

### Special details

**Table:**

Geometry. All e.s.d.'s (except the e.s.d. in the dihedral angle between two l.s. planes) are estimated using the full covariance matrix. The cell e.s.d.'s are taken into account individually in the estimation of e.s.d.'s in distances, angles and torsion angles; correlations between e.s.d.'s in cell parameters are only used when they are defined by crystal symmetry. An approximate (isotropic) treatment of cell e.s.d.'s is used for estimating e.s.d.'s involving l.s. planes.

### Fractional atomic coordinates and isotropic or equivalent isotropic displacement parameters (Å^2^)

**Table:**

	*x*	*y*	*z*	*U*_iso_*/*U*_eq_	
Cu1	1.0000	0.48258 (3)	0.2500	0.03297 (12)	
F1	0.92606 (7)	0.77593 (17)	0.00333 (8)	0.0623 (4)	
F2	0.68438 (9)	0.54002 (17)	0.09264 (9)	0.0671 (4)	
O1	0.92455 (8)	0.61557 (16)	0.16170 (8)	0.0455 (3)	
O2	0.86509 (13)	0.40923 (19)	0.09534 (10)	0.0696 (5)	
N1	1.10527 (11)	0.49323 (17)	0.19221 (10)	0.0389 (4)	
N2	1.0000	0.2460 (2)	0.2500	0.0373 (5)	
C1	0.87159 (13)	0.5462 (2)	0.10665 (11)	0.0426 (4)	
C2	0.80884 (12)	0.6517 (2)	0.05110 (11)	0.0380 (4)	
C3	0.83842 (11)	0.7617 (2)	0.00333 (11)	0.0405 (4)	
C4	0.71868 (13)	0.6428 (2)	0.04644 (11)	0.0421 (4)	
C5	1.11090 (14)	0.6002 (2)	0.13562 (13)	0.0497 (5)	
H5	1.0606	0.6562	0.1145	0.060*	
C6	1.18819 (17)	0.6303 (3)	0.10757 (16)	0.0629 (6)	
H6	1.1899	0.7058	0.0685	0.076*	
C7	1.26229 (16)	0.5481 (3)	0.13773 (16)	0.0648 (7)	
H7	1.3154	0.5678	0.1202	0.078*	
C8	1.25719 (14)	0.4362 (3)	0.19412 (16)	0.0627 (6)	
H8	1.3065	0.3770	0.2144	0.075*	
C9	1.17809 (13)	0.4123 (3)	0.22054 (13)	0.0497 (5)	
H9	1.1753	0.3371	0.2595	0.060*	
C10	1.00346 (13)	0.1681 (2)	0.18168 (13)	0.0462 (5)	
H10	1.0056	0.2218	0.1337	0.055*	
C11	1.00405 (16)	0.0110 (3)	0.1796 (2)	0.0640 (7)	
H11	1.0071	−0.0405	0.1312	0.077*	
C12	1.0000	−0.0680 (4)	0.2500	0.0727 (11)	
H12	1.0000	−0.1741	0.2500	0.087*	

### Atomic displacement parameters (Å^2^)

**Table:**

	*U*^11^	*U*^22^	*U*^33^	*U*^12^	*U*^13^	*U*^23^
Cu1	0.03026 (18)	0.03081 (18)	0.03662 (19)	0.000	0.00260 (12)	0.000
F1	0.0316 (6)	0.0927 (10)	0.0611 (8)	0.0037 (6)	0.0038 (5)	0.0250 (7)
F2	0.0513 (7)	0.0780 (9)	0.0705 (9)	−0.0032 (7)	0.0064 (6)	0.0393 (7)
O1	0.0377 (7)	0.0536 (8)	0.0402 (7)	0.0054 (6)	−0.0064 (6)	0.0110 (6)
O2	0.0893 (12)	0.0491 (10)	0.0593 (10)	0.0198 (8)	−0.0170 (9)	0.0028 (7)
N1	0.0353 (8)	0.0404 (8)	0.0402 (9)	−0.0013 (6)	0.0049 (7)	−0.0037 (6)
N2	0.0348 (10)	0.0303 (10)	0.0452 (12)	0.000	0.0033 (9)	0.000
C1	0.0415 (10)	0.0502 (12)	0.0338 (10)	0.0121 (9)	0.0004 (8)	0.0079 (8)
C2	0.0375 (9)	0.0422 (10)	0.0312 (9)	0.0068 (7)	−0.0024 (7)	0.0030 (7)
C3	0.0291 (8)	0.0537 (11)	0.0364 (9)	0.0033 (8)	−0.0004 (7)	0.0041 (8)
C4	0.0419 (10)	0.0468 (11)	0.0359 (9)	0.0003 (8)	0.0024 (8)	0.0108 (8)
C5	0.0488 (11)	0.0494 (12)	0.0520 (12)	−0.0007 (9)	0.0121 (9)	0.0049 (9)
C6	0.0667 (15)	0.0669 (15)	0.0601 (14)	−0.0140 (12)	0.0245 (12)	0.0020 (11)
C7	0.0447 (12)	0.0851 (17)	0.0687 (16)	−0.0165 (12)	0.0215 (11)	−0.0195 (14)
C8	0.0354 (11)	0.0820 (17)	0.0692 (15)	0.0034 (11)	0.0052 (10)	−0.0112 (14)
C9	0.0380 (10)	0.0579 (13)	0.0512 (12)	0.0037 (9)	0.0025 (9)	−0.0002 (10)
C10	0.0416 (10)	0.0398 (10)	0.0560 (12)	0.0010 (8)	0.0053 (9)	−0.0104 (9)
C11	0.0498 (13)	0.0455 (13)	0.094 (2)	0.0042 (9)	0.0057 (13)	−0.0265 (12)
C12	0.0525 (19)	0.0297 (15)	0.132 (4)	0.000	0.007 (2)	0.000

### Geometric parameters (Å, °)

**Table:**

Cu1—N1	2.0236 (16)	C4—C3^ii^	1.376 (3)
Cu1—N1^i^	2.0236 (16)	C5—C6	1.376 (3)
Cu1—O1	2.0609 (12)	C5—H5	0.9300
Cu1—O1^i^	2.0609 (12)	C6—C7	1.365 (4)
Cu1—N2	2.073 (2)	C6—H6	0.9300
F1—C3	1.352 (2)	C7—C8	1.368 (4)
F2—C4	1.350 (2)	C7—H7	0.9300
O1—C1	1.266 (2)	C8—C9	1.379 (3)
O2—C1	1.216 (3)	C8—H8	0.9300
N1—C9	1.337 (3)	C9—H9	0.9300
N1—C5	1.341 (3)	C10—C11	1.377 (3)
N2—C10	1.333 (2)	C10—H10	0.9300
N2—C10^i^	1.333 (2)	C11—C12	1.370 (4)
C1—C2	1.523 (2)	C11—H11	0.9300
C2—C4	1.375 (3)	C12—C11^i^	1.371 (4)
C2—C3	1.376 (3)	C12—H12	0.9300
C3—C4^ii^	1.376 (3)		
			
N1—Cu1—N1^i^	174.71 (9)	F2—C4—C3^ii^	118.40 (17)
N1—Cu1—O1	91.84 (6)	C2—C4—C3^ii^	121.81 (17)
N1^i^—Cu1—O1	85.16 (6)	N1—C5—C6	122.6 (2)
N1—Cu1—O1^i^	85.17 (6)	N1—C5—H5	118.7
N1^i^—Cu1—O1^i^	91.84 (6)	C6—C5—H5	118.7
O1—Cu1—O1^i^	111.11 (8)	C7—C6—C5	119.2 (2)
N1—Cu1—N2	92.64 (4)	C7—C6—H6	120.4
N1^i^—Cu1—N2	92.64 (4)	C5—C6—H6	120.4
O1—Cu1—N2	124.45 (4)	C6—C7—C8	119.0 (2)
O1^i^—Cu1—N2	124.44 (4)	C6—C7—H7	120.5
C1—O1—Cu1	116.73 (12)	C8—C7—H7	120.5
C9—N1—C5	117.60 (18)	C7—C8—C9	119.2 (2)
C9—N1—Cu1	119.83 (14)	C7—C8—H8	120.4
C5—N1—Cu1	121.40 (14)	C9—C8—H8	120.4
C10—N2—C10^i^	118.4 (2)	N1—C9—C8	122.4 (2)
C10—N2—Cu1	120.81 (12)	N1—C9—H9	118.8
C10^i^—N2—Cu1	120.81 (12)	C8—C9—H9	118.8
O2—C1—O1	127.60 (17)	N2—C10—C11	122.4 (2)
O2—C1—C2	118.69 (17)	N2—C10—H10	118.8
O1—C1—C2	113.70 (17)	C11—C10—H10	118.8
C4—C2—C3	116.07 (16)	C12—C11—C10	118.8 (3)
C4—C2—C1	121.48 (17)	C12—C11—H11	120.6
C3—C2—C1	122.45 (16)	C10—C11—H11	120.6
F1—C3—C2	119.69 (16)	C11—C12—C11^i^	119.3 (3)
F1—C3—C4^ii^	118.18 (17)	C11—C12—H12	120.4
C2—C3—C4^ii^	122.12 (17)	C11^i^—C12—H12	120.4
F2—C4—C2	119.78 (17)		
			
N1—Cu1—O1—C1	98.39 (14)	O2—C1—C2—C3	−120.1 (2)
N1^i^—Cu1—O1—C1	−85.98 (14)	O1—C1—C2—C3	61.3 (2)
O1^i^—Cu1—O1—C1	−176.08 (15)	C4—C2—C3—F1	−178.79 (17)
N2—Cu1—O1—C1	3.92 (15)	C1—C2—C3—F1	1.7 (3)
O1—Cu1—N1—C9	−174.91 (15)	C4—C2—C3—C4^ii^	−0.2 (3)
O1^i^—Cu1—N1—C9	74.06 (15)	C1—C2—C3—C4^ii^	−179.68 (18)
N2—Cu1—N1—C9	−50.30 (15)	C3—C2—C4—F2	−178.63 (18)
O1—Cu1—N1—C5	17.73 (16)	C1—C2—C4—F2	0.9 (3)
O1^i^—Cu1—N1—C5	−93.30 (16)	C3—C2—C4—C3^ii^	0.2 (3)
N2—Cu1—N1—C5	142.34 (15)	C1—C2—C4—C3^ii^	179.68 (18)
N1—Cu1—N2—C10	−49.29 (11)	C9—N1—C5—C6	−1.2 (3)
N1^i^—Cu1—N2—C10	130.71 (11)	Cu1—N1—C5—C6	166.42 (18)
O1—Cu1—N2—C10	44.76 (11)	N1—C5—C6—C7	0.5 (4)
O1^i^—Cu1—N2—C10	−135.25 (11)	C5—C6—C7—C8	1.0 (4)
N1—Cu1—N2—C10^i^	130.71 (11)	C6—C7—C8—C9	−1.7 (4)
N1^i^—Cu1—N2—C10^i^	−49.29 (11)	C5—N1—C9—C8	0.5 (3)
O1—Cu1—N2—C10^i^	−135.24 (11)	Cu1—N1—C9—C8	−167.33 (18)
O1^i^—Cu1—N2—C10^i^	44.76 (11)	C7—C8—C9—N1	0.9 (4)
Cu1—O1—C1—O2	−6.9 (3)	C10^i^—N2—C10—C11	−0.36 (16)
Cu1—O1—C1—C2	171.55 (12)	Cu1—N2—C10—C11	179.65 (16)
O2—C1—C2—C4	60.4 (3)	N2—C10—C11—C12	0.7 (3)
O1—C1—C2—C4	−118.2 (2)	C10—C11—C12—C11^i^	−0.33 (15)

Symmetry codes: (i) −*x*+2, *y*, −*z*+1/2; (ii) −*x*+3/2, −*y*+3/2, −*z*.
